# NURR1 Deficiency Is Associated to Altered Microglial Phenotype in Male Mice

**DOI:** 10.1007/s12035-025-04787-8

**Published:** 2025-03-08

**Authors:** Francesca Montarolo, Sarah Thielens, Maria Bove, Antonio Bertolotto, Filippo Tempia, Eriola Hoxha

**Affiliations:** 1 Department of Neurosciences “Rita Levi Montalcini”, University of Torino, Turin, Italy; 2 Neuroscience Institute Cavalieri Ottolenghi (NICO), Orbassano, TO Italy; 3 Department of Neurosciences, University of Mons, Mons, Belgium; 4 Department of Clinical and Experimental Medicine, University of Foggia, Foggia, Italy; 5 Koelliker Hospital, Turin, Italy

**Keywords:** NURR1, Microglia, Hyperactive behaviour, Midbrain, Substantia nigra (SN)

## Abstract

The transcription factor NUclear Receptor Related 1 (NURR1) regulates the development and maintenance of midbrain dopaminergic (mDA) neurons, which control voluntary movement, motivation, and reward. NURR1 also plays anti-inflammatory functions in microglia, protecting mDA neurons from inflammation-induced death. It remains to be determined to what extent NURR1 exerts its function in microglia. Interestingly, altered microglial phenotypes are associated to psychiatric conditions. NURR1 defects in male mice are associated with hyperactive and impulsive behaviour. Notably, such behaviour is accompanied by a normal development of mDA neurons which, at least in their number, are preserved. This study aims to explain the altered behaviour of NURR1-deficient mice by analyzing microglial compartment and inflammatory machinery that could be consistently altered to influence such observed behaviours. The present work demonstrates that NURR1 deficiency determines a reduction in the number of microglial cells specifically in the substantia nigra (SN), without altering their morphological activation state. Gene expression levels of molecules associated with active/protective microglial phenotype in the SN of NURR1^+/−^ mice are altered. The level of HMOX, a marker of cellular damage/apoptosis, is up-regulated, while the level of MT2, a marker of response to stress, is reduced in the SN of NURR1^+/−^ mice. The level of prostaglandin receptors, which are endogenous ligands for NURR1, is up-regulated in the same compartment. Overall, the NURR1-deficient mice, which exhibit impaired behaviour, have a reduced number of microglia cells and alterations of the inflammatory machinery in their SN.

**Supplementary Information:**

The online version contains supplementary material available at 10.1007/s12035-025-04787-8.

## Introduction

The protein NUclear Receptor Related 1 (NURR1, also called NR4A2) is a transcription factor belonging to the family of the steroid hormone receptor. Although NURR1 shares typical features of steroid hormone receptor organization including an N-terminal transactivation domain, a DNA-binding domain and a C-terminal ligand-binding domain (LBD), there are limited studies about endogenous ligands that regulate its transcriptional activity [1]. To date, only lipid-mediated metabolites, such as prostaglandins, have been found to directly interact with the LBD of NURR1 to stimulate its receptor functions [2].

In the central nervous system (CNS), NURR1 is required for the development and functioning of the midbrain dopaminergic (mDA) neurons [3, 4], which are a class of ventral mesencephalic neurons critical for controlling voluntary movement, motivating behaviour, reward, and emotion [5]. Indeed, the complete NURR1 ablation results in the full agenesis of the mDA neurons [4]. NURR1 generates and maintains the mDA neurons controlling the transcription of the dopamine (DA) signalling pathway genes, such as tyrosine hydroxylase (TH), DA transporter 1 (DAT1), and vesicular monoamine transporter 2 (VMAT2) [4, 6, 7]. Hence, polymorphisms and mutations resulting in reduced expression of NURR1 are associated with familial and sporadic Parkinson’s disease (PD), where selective degeneration of mDA neurons occurs [8–12]. A growing number of studies also reported promising data concerning the NURR1 activation in PD models able to protect mDA neurons from neurotoxicity and motor behaviours associated with DA neurotransmission [2, 13–15]. Dopaminergic signalling is also altered in psychiatric disorders and behaviours, such as schizophrenia [16] and addiction [17]; thus, altered NURR1 expression is also implicated in such diseases [18, 19].

While the role of NURR1 in mDA neurons is widely studied, recent evidence indicates that NURR1 also plays an important role in neuro-protection mediated by its anti-inflammatory functions [20–23]. In vitro, NURR1 inhibits the expression of pro-inflammatory mediators mainly in microglia, and accordingly, it protects mDA neurons from inflammation-induced death [24]. However, the exact mechanism by which NURR1 may influence the microglial phenotype is not yet understood.

Numerous evidence clearly report that microglia, which are resident phagocytic cells of CNS, are not only involved in immune response, but also in the control of brain tissue homeostasis influencing neuro-inflammatory features in health and disease [25]. Microglia during development and adulthood continuously scan their surrounding environments by modifying their morphology. The morphology of microglia changes from a resting state characterized by small soma size with longer and ramified branches, to an activated amoeboid state with an increased soma size and shorter and thicker branches [26, 27]. These morphologies are closely related to their functional state [26]. In the resting state microglia prune synapses and regulate neuronal activity, acting on neurotransmitter signalling and synaptic transmission [28–31]. In the activated state, microglia acquire phagocytic function starting also to produce effector molecules to induce a pro- or anti-inflammatory features related to a damaging or restorative phenotype [32, 33]. The changes allow to consider the ratio between the soma size and cell area as a parameter of their activation state [33]. Accumulating studies indicate that altered microglial phenotypes are associated to psychiatric and neurodegenerative conditions, such as depression [34], Alzheimer’s disease [35], and PD [36].

The beneficial role of NURR1 in the CNS has been also related to its capacity to protect neurons against the detrimental effects of reactive oxygen species (ROS) [37] that are able to induce lipid peroxidation. Indeed, it has been shown that neuronal cells are highly vulnerable to oxidative damage because of their high oxygen consumption, weak antioxidant defence, and high polyunsaturated fatty acid (PUFA) content in membranes [38]. Malondialdehyde (MDA) is one of the final products of PUFA peroxidation, thus being considered a reliable indirect marker of oxidative stress [39]. Moreover, NURR1 has been described to protect mDA neurons by increasing expression levels of anti-oxidant proteins and, therefore, counteracting the imbalance of the redox system [40]. In this regard, the administration of a NURR1 agonist was found to exert a neuroprotective role in an oxidative stress-induced rodent model of PD [41].

In the past, the NURR1-deficient mouse was suggested as a model for DA-associated brain disorders, including PD [42] and schizophrenia [43], generating some confusion. However, recently our studies, [44, 45] in agreement with published works [42, 43, 46, 47], highlighted its restricted behavioural phenotype. In detail, only adult male heterozygous NURR1-knockout (NURR1^+/−^) mice are both hyperactive and impulsive without alterations in motor coordination, anxiety, sociability, or memory [44]. On the contrary, female NURR1^+/−^ mice are only impulsive [45]. The behaviour phenotype, however, is accompanied by a normal development of mDA neurons which, at least in their number and in the gene expression level of TH, are preserved both in the mesencephalic substantia nigra (SN) and ventral tegmental area (VTA) [44]. Also, the level of DA in brain and plasma of NURR1^+/−^ mice is conserved [44].

The central hypothesis is that the behavioural alterations of male NURR1^+/−^ mice might be due to changes in microglial function and activation of inflammatory pathways. Therefore, the aim of this work is to search for changes in microglial function, which might be related to a cerebral tissue inflammatory process.

## Materials and Methods

### Animals

Three to five-month-old male NURR1^+/−^ and their wild-type (WT) littermates were used for all experimental paradigms. The NURR1^+/−^ mice were obtained from Prof. Orla M. Conneely, Baylor College of Medicine, Houston, USA. Since homozygous NURR1-knockout mice die within 12 h after birth [4], heterozygous (NURR1^+/−^) mice were used. Their genotype was confirmed by means of polymerase chain reaction (PCR), as reported in [44, 45, 48] and in the Supplementary material and methods section. All experimental procedures were carried out at the Neuroscience Institute Cavalieri Ottolenghi (NICO), approved by the Ethical Committee of the University of Torino and authorized by the Italian Ministry of Health (authorization number: 56/2017-PR and 586/2020). The experiments have been carried out in accordance with the European Communities Parliament and Council Directives of 24 November 1986 (86/609/EEC) and 22 September 2010 (2010/63/EU). Mice were housed with a 12 h light/dark cycle and had free access to food/water. Adequate measures were taken to minimize pain and discomfort.

### Histological Procedures

Mice (WT, *n* = 7, and NURR1^+/−^, *n* = 6) were deeply anesthetized (ketamine 200 mg/kg, xylazine 50 mg/kg) and trans-cardially perfused with 4% paraformaldehyde in 0.12 M phosphate buffer, pH 7.2–7.4. The brains were removed and immersed in the same fixative at 4 °C for 24 h and then cryo-protected in 30% sucrose in 0.12 M phosphate buffer. Brains were frozen and serially cut by a cryostat in 30-μm-thick coronal sections collected in phosphate buffered saline (PBS). In order to detect ionized calcium-binding adaptor molecule 1 positive (Iba1+) microglial cells and glial fibrillary acidic protein positive (GFAP+) astrocytes, slices were incubated overnight at 4 °C with the polyclonal anti-rabbit Iba1 (019–19741, Wako Chemicals, Richmond, VA) and the polyclonal anti-rabbit GFAP (Z033429-2, Dako, Agilent, Santa Clara, CA) diluted 1:1000 and 1:500, respectively in PBS with 1% TritonX-100 and 1.5% normal donkey serum. Immunohistochemical reactions were performed by the avidin–biotin–peroxidase method (Vectastain ABC Elite kit; Vector Laboratories, Burlingame, CA, USA) and revealed using 3,3′-diaminobenzidine (3% in Tris–HCl) as chromogen as reported in [44, 49]. After processing, sections were mounted on microscope slides with Neo Mount (1.09016, Merck, Darmstadt, Germany). In order to detect the morphology of Iba1 + cells, immunofluorescence staining was performed as follow (WT, *n* = 5, and NURR1^+/−^, *n* = 5). Incubation with primary antibody anti-Iba1 (019–19741, Wako Chemicals, Richmond, VA) was made overnight at 4 °C in PBS with 1% Triton-X 100 and 1.5% normal donkey serum. The sections were then exposed for 2 h at room temperature with secondary anti-rabbit Cy3-conjugated antibody (Jackson ImmunoResearch Laboratories, West Grove, PA). 4,6-diamidino-2- phenylindole (DAPI, Fluka, Saint Louis, USA) was used to counterstain cell nuclei. Sections were mounted on microscope slides with Tris-glycerol supplemented with 10% Mowiol (Calbiochem, LaJolla, CA).

### Image Acquisition and Processing

Images from histological specimens were acquired by means of the ZEISS Axioscan 7 Brightfield microscope slide scanner (Oberkochen, Germany) and the Leica TCS SP5 confocal microscope. All images were evaluated by means of the ImageJ software (http://rsbweb.nih.gov/ij/index.html). To recognize the mouse brain regions the Paxinos and Franklin Mouse Brain Atlas was used [50]. Random frame of the substantia nigra (SN) and ventral tegmental area (VTA) were acquired within the stereotaxic coordinates from Bregma − 2.80 mm, Interaural 1.00 to Bregma − 3.88 mm, Interaural − 0.08. Random frame of the nucleus accumbens (NAc) and caudate putamen (CPu) was acquired within the stereotaxic coordinates from Bregma 1.70 mm, Interaural 5.50 to Bregma 0.62 mm, Interaural 4.42.

ZEISS Axioscan brightfield images were captured with a Plan Apochromat 20X/0.8 M27 objective, and with exposure time of 200 us. The number of Iba1+ microglia and GFAP+ astrocyte was calculated as number of cell per mm^2^ (cell number/mm^2^). At least two images (i.e., one random frame in the right hemisphere and one in the left hemisphere) were analysed for each of the three selected brain slices.

Confocal images were captured as z-stacked focal planes through the thickness of the slice (30 μm) at 0.5-μm optical steps with an oil-immersed Plan-Apochromat 40X/1.25 objective, zoom 1.0, and resolution of 1024/1024 pixels and 100 Hz (1 pixel = 0.38 μm) in SN. The morphology of the Iba1+ microglial cells was reconstructed through the Simple Neural Tracer (SNT) plugins of ImageJ software in confocal immunofluorescent images, as reported in [27, 51] and in Supplementary Fig. 1. For each animal four Iba1 + cells were reconstructed, resulting in 20 cells for the five WT and the five NURR1^+/−^ mice. From the reconstructed morphology, the total processes length (i.e., the sum of length of all the reconstructed processes per each cell), the total number of branches (i.e., the sum of all reconstructed branches per each cell), and the total number of intersection of the Sholl analysis (i.e., the number of intersections between branches and each concentric circles at specified radii (2 µm) centred on the microglial soma per each cell) were evaluated. The cell area (i.e., area occupied by the whole cell including both soma and cell processes) and the cell perimeter (i.e., perimeter of the whole cell including both soma and cell processes) were obtained by analysing the binary mask image of the reconstructed Iba1 + cells as reported in [33]. The territory area was obtained using the binary mask image and tracking the smallest convex polygon (internal angles smaller than 180°) in which the reconstructed Iba1 + cell was inscribed as reported in [33]. The territory area is the area of the drawn polygon, while the convex hull perimeter is the perimeter of such polygon. The soma size of the Iba1 + microglial cells reported in µm^2^ was evaluated tracking the cellular area.

### Substantia Nigra (SN) Collection

Mice (WT, *n* = 15, and NURR1^+/−^, *n* = 11) were euthanized by inhalation of isoflurane, and brains were removed. The SN (Bregma − 2.80 mm, Interaural 1.00; Bregma − 3.88 mm, Interaural − 0.08) was manually dissected from 300-μm-thick coronal sections vibratome and brain matrix to perform semiquantitative RT real-time PCR analysis and ROS/MDA measurement, respectively. All samples were rapidly frozen in 2-methylbutane in dry ice and stored at − 80 °C.

## Semiquantitative RT Real-Time PCR Analysis

Total RNA (WT, *n* = 8, and NURR1^+/−^, *n* = 4) was isolated by extraction with the Pure Link RNA Mini Kit (Thermo Fisher Scientific, USA) according to the manufacturer’s instructions and as reported in [52]. Total RNA was reverse-transcribed to complementary DNA (cDNA) at a final concentration of 20 ng/μl using the High Capacity Kit (Thermos Fisher Scientific, Waltham, MA, USA). Gene expression analysis was performed by real-time PCR using Applied Biosystems’ TaqMan gene expression products (Thermos Fisher Scientific, Waltham, MA, USA). Transcriptional expression was normalized using glyceraldehyde-3-phosphate dehydrogenase (GAPDH) as reference gene. For primers and probes, Applied Biosystems’ TaqMan® Assay-on-demand-TM gene expression products were used. Expression levels of target genes were calculated by the normalized comparative cycle threshold (Ct) method (2^−DCt^).

## Reactive Oxygen Species (ROS) Measurement

ROS measurement in SN (WT, *n* = 7, and NURR1^+/−^, *n* = 7) was performed as previously described [53–55] by using the fluorogenic dye 2′,7′dichlorofluorescein diacetate (Sigma-Aldrich, Milan, Italy) [56, 57]. Briefly, tissue was homogenized in PBS (pH = 7.4), according to the following proportion: 500 μl of PBS for 2.5 mg of tissue. The dye was added to the sample with a final concentration of 5 mM, and incubation was performed for 15 min at 37 °C. Samples were than centrifuged for 10 min at 4 °C and 12,500 rpm. The pellet was suspended in 5 ml PBS and put in ice for 10 min. After incubation of 1 h at 37 °C, samples were analysed in a 96-well microplate by using a fluorometer (Filter Max F5, Multi-Mode Microplate Reader, excitation length 475 nm, emission length 535 nm). Results were expressed as μmol DCF/mg of tissue.

### Malondialdehyde (MDA) Assay

MDA assay in SN (WT, *n* = 7, and NURR1^+/−^, *n* = 7) was performed by using a commercially available kit (Sigma-Aldrich, Milan, Italy) as previously described [56, 58], according to the manufacturer’s instructions. Each sample and standard analysis was performed in duplicate to avoid intra-assay variations.

### Statistical Analysis

Statistical analyses performed by means of Shapiro–Wilk test to assess distribution of samples, Mann–Whitney *U* test, and two-way analysis of variance (ANOVA) were carried out by GraphPad Prism 8 (GraphPad Software Inc., La Jolla, CA, USA). Statistical significance was considered at *p* values < 0.05.

## Results

### *NURR1*^+*/−*^* Mice Show a Reduced Number of Iba1* + *Microglial Cells in SN Compared to WT Mice*

**Fig. 1:**
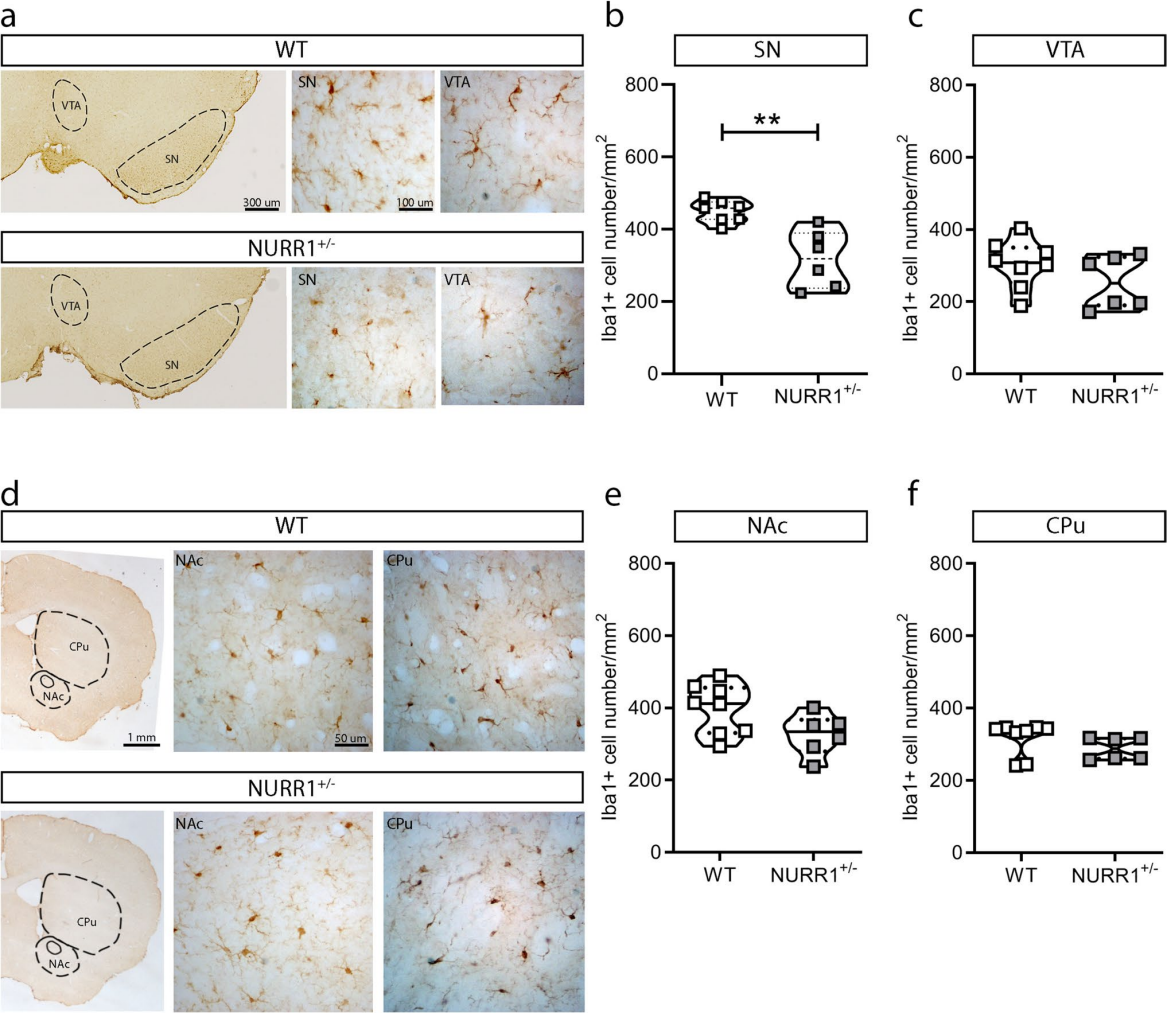
**NURR1**^+/−^**mice show a reduction in the number of Iba1+ microglial cells in the SN compared to WT mice. a** Representative images of coronal brain sections of WT and NURR1^+/−^ mice stained with anti-Iba1 antibody at 1× and 20× magnification. The sections highlight the substantia nigra (SN) and the ventral tegmental area (VTA) regions. **b**, **c** Quantitative analysis discloses a reduction in number of Iba1+ microglial cells in the SN (**b**), but not in the VTA (**c**) of NURR1^+/−^ mice (*n* = 6) compared to WT (*n* = 7). **d** Representative images of coronal brain sections of WT and NURR1^+/−^ mice stained with anti-Iba1 antibody at 1× and 20× magnification. The sections highlight the nucleus accumbens (NAc) and the caudate putamen (CPu). **e**, **f** Quantitative analysis discloses no differences between genotypes (WT *n* = 7; NURR1^+/−^ *n* = 6) in the number of Iba1+ microglial cells in NAc (**e**) and CPu (**f**). Mann–Whitney *U* test, **p < 0.01

To dissect the possible impact of NURR1 deficiency on the microglial cells, the Iba1+ cell number was calculated in the areas implied in the dopaminergic pathway such as SN, VTA, and in their target regions, such as NAc and CPu. NURR1^+/−^ mice show a reduction in the Iba1+ cell number in SN (Fig. 1a, b, ** *p* = 0.0023, Mann–Whitney *U* test), but not in VTA (Fig. 1a, c, *p* = 0.3450, Mann–Whitney *U* test), NAc (Fig. 1d, e, *p* = 0.0813, Mann–Whitney *U* test), and CPu (Fig. 1d, f, *p* = 0.1419, Mann–Whitney *U* test) compared to their WT littermates.

Considering the possible role that NURR1 could exert in the astrocyte compartment, also the GFAP+ astrocyte cell number was measured in the same areas. No differences emerged in this parameter between genotypes in each of the analysed regions (Supplementary Fig. 2, for SN *p* = 0.3450 (a), for VTA *p* = 0.7796 (b), for NAc *p* = 0.6889 (c), for CPu *p* = 0.4559 (d), Mann–Whitney *U* test).

### *NURR1*^+*/−*^* Mice Show Conserved Morphology of Iba1*+ *Microglial Cells Compared to WT Mice*

**Fig. 2:**
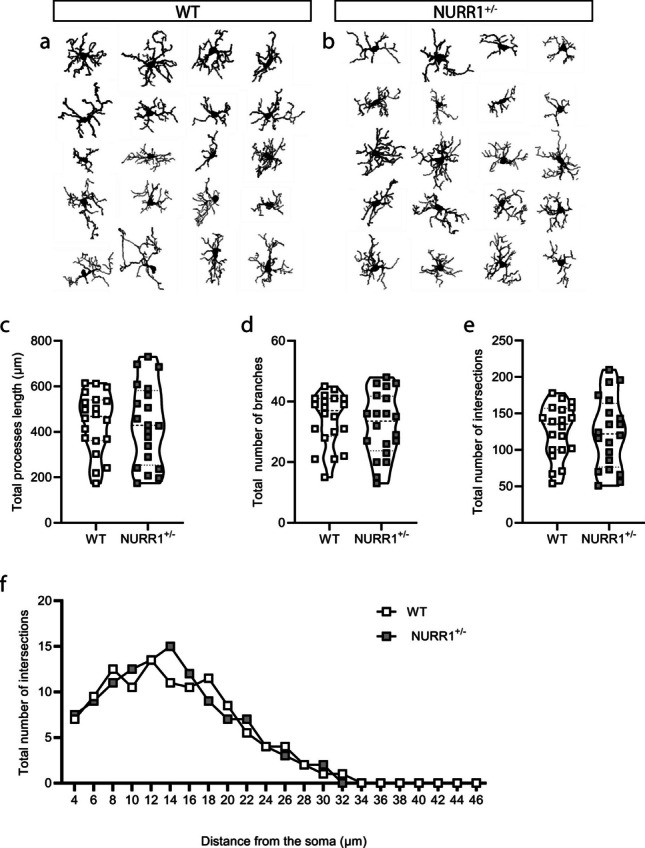
**NURR1**^+/−^** mice show no alterations in the branches of the Iba1+ microglial cells compared to WT mice.** (**a**, **b**) Morphological reconstruction of the Iba1+ cells of the SN of WT (**a**) and NURR1^+/−^ (**b**) mice. Each row represents the 4 reconstructed cells for each mouse. Quantitative analysis of the total processes length (**c**), and the total number of branches (**d**), reveals no differences between genotypes. Sholl analysis reported as the total number of intersections (**e**), and as the function of distance from the soma (**f**) reveals no differences between genotypes. Mann-Whitney U test and two-way ANOVA. (WT *n*=20 cells obtained from 5 mice; NURR1^+/−^ *n*=20 cells obtained from 5 mice)

To better understand whether the reduced number of microglial cells observed in the SN of NURR1^+/−^ mice is accompanied by a change in their phenotype, a detailed analysis on the Iba1+ cell morphology was conducted using the immunofluorescence images acquired with confocal microscopy. Specifically, the morphological reconstruction of the Iba1+ cell in SN is reported for WT (Fig. 2a) and NURR1^+/−^ (Fig. 2b) mice. The reduction in the number of Iba1+ microglial cells in SN of NURR1^+/−^ mice compared to WT was confirmed also in these sets of acquired images (data not shown).

No differences emerge in NURR1^+/−^ mice in the total processes length (Fig. 2c, *p* = 0.7994, Mann–Whitney *U* test) or, in the total number of branches (Fig. 2d, *p* = 0.6152, Mann–Whitney *U* test) compared to WT.

An additional fine analysis to further examine the complexity of the Iba1+ cellular processes was performed using the Sholl analysis. No differences emerge in NURR1^+/−^ mice compared to WT mice, considering the intersections between branches and concentric circles measured as total number (Fig. 2e, *p* = 0.7532, Mann–Whitney *U* test), and as function of distance from the soma (Fig. 2f, two-way ANOVA, distance from the soma: *F* (24, 950) = 89.46, *p* < 0.0001, genotype: *F* (1, 950) = 0.1158, *p* = 0.7337).

**Fig. 3:**
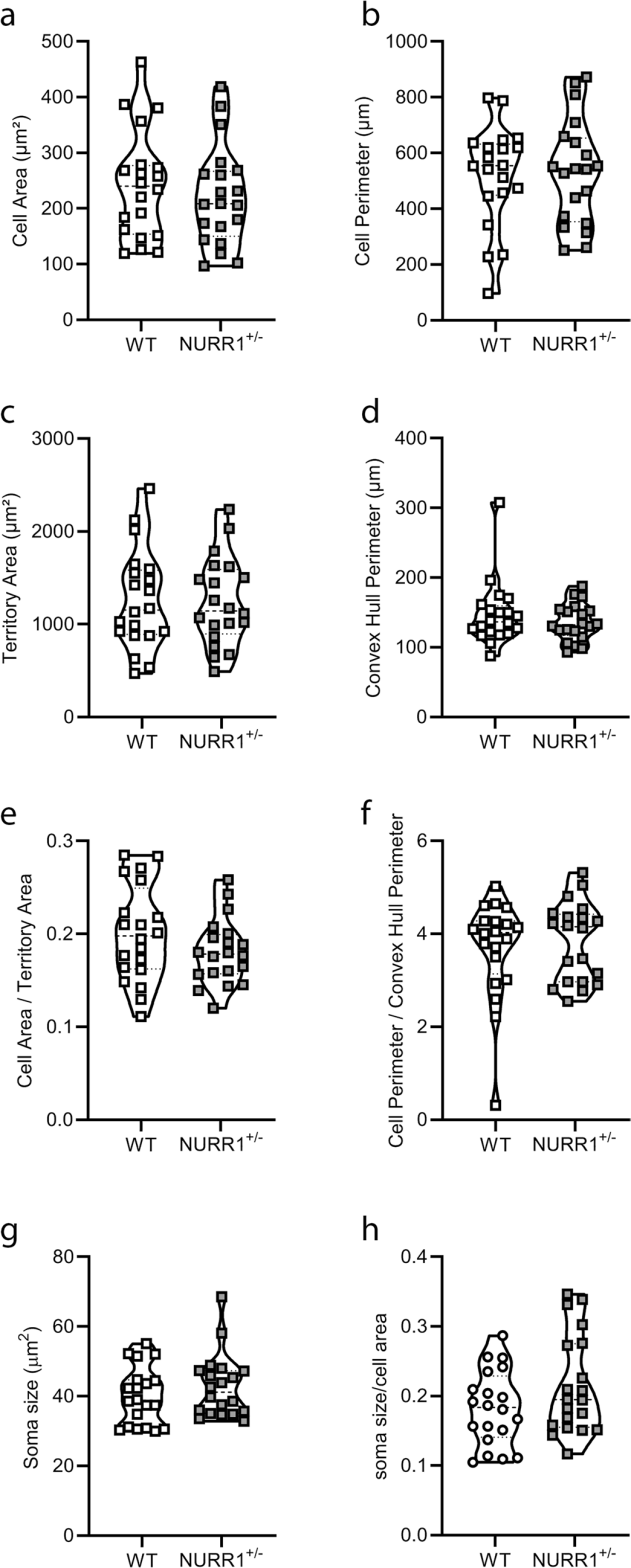
**NURR1**^+/−^** mice show no alterations in the morphology of Iba1 + microglial cells compared to WT mice.** Quantitative analysis of the cell area (**a**), cell perimeter (**b**), territory area (**c**), convex hull perimeter (**d**), ratio between cell area and territory area (**e**), ratio between cell perimeter and convex hull perimeter (**f**), soma size (**g**), and ratio between soma size and cell area (**h**) reveals no differences between genotypes. Mann–Whitney *U* test. (WT *n* = 20 cells obtained from five mice; NURR1^+/−^ *n* = 20 cells obtained from five mice)

The morphologically reconstructed Iba1+ cells were further evaluated, and no differences emerge in NURR1^+/−^ mice regarding their cell area (Fig. 3a, *p* = 0.4777, Mann–Whitney *U* test), cell perimeter (Fig. 3b, *p* = 0.9838, Mann–Whitney *U* test), territory area (Fig. 3c, *p* = 0.9254, Mann–Whitney *U* test), and convex hull perimeter (Fig. 3d, *p* = 0.6588, Mann–Whitney *U* test) compared to WT. Also, the analysis carried out on the ratio between cell area and territory area (Fig. 3e, *p* = 0.2012, Mann–Whitney *U* test), and between cell perimeter and convex hull perimeter (Fig. 3f, *p* = 0.7788, Mann–Whitney *U* test) relating to a possible index of microglial activation, shows no differences in NURR1^+/−^ compared to WT mice. To further confirm the results of the absence of an altered activated state in the Iba1 + cells, also the soma size (Fig. 3g) and the ratio between soma size and cell area (Fig. 3h) were measured. No differences emerge in NURR1^+/−^ mice compared to WT (for soma size *p* = 0.3107, for ratio *p* = 0.2012, Mann–Whitney *U* test), indicating that the Iba1 + microglial cells are not differently activated compared to WT.

### *NURR1*^+*/−*^* Mice Show Alterations in the Gene Expression Level of Effector Molecules Related to the Active and Neuroprotective Microglial Phenotype*

In order to characterize the environment in which we observe the reduction of the Iba1+ microglial cell number without differences in their activated state, a gene expression analysis in the SN of NURR1^+/−^ and WT mice was conducted.

No significant differences emerge between genotype in the expression of the DAT1, DA receptor 1 (DRD1), and DA receptor 2 (DRD2) (Supplementary Fig. 3, for DAT1 *p* = 0.4121 (a), for DRD1 *p* = 0.5273 (b), for DRD2 *p* = 0.9273 (c), Mann–Whitney *U* test), indicating that the SN of NURR1^+/−^ mice is not characterized by DA alteration.

**Fig. 4:**
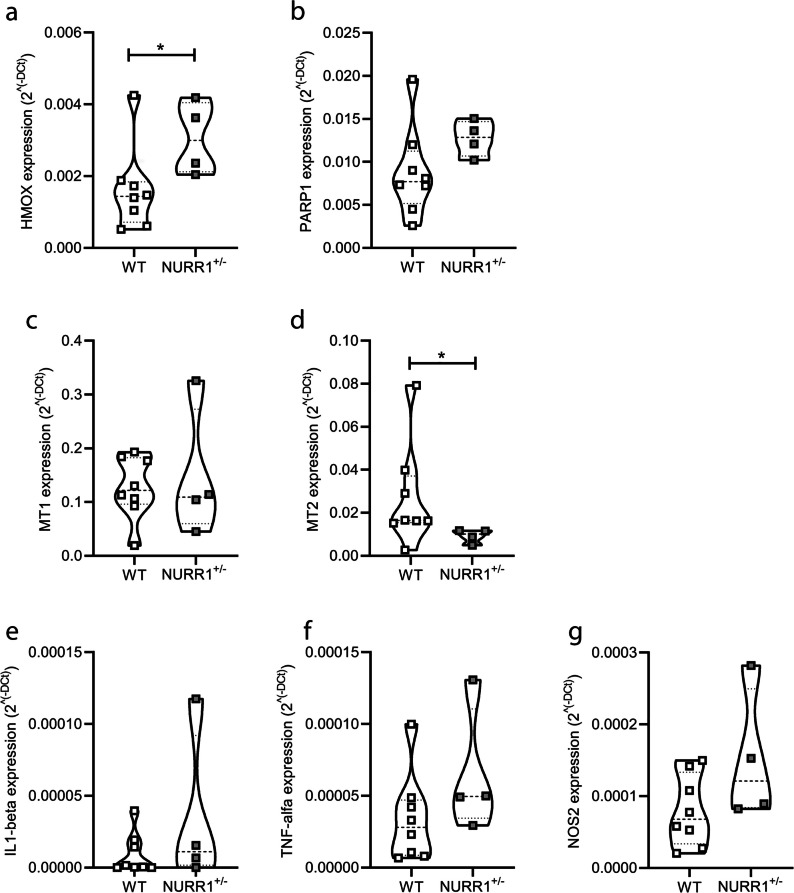
**Expression levels of effector molecules related to the active microglial phenotype**. Comparison of gene expression levels in the SN of WT (*n* = 8) and NURR1^+/−^ (*n* = 4) mice of the heme oxygenase 1 (HMOX) (**a**), the poly ADP-ribose polymerase family, member 1 (PARP1) (**b**), the metallothionein 1 (MT1) (**c**), the metallothionein 2 (MT2) (**d**), the interleukin-1-beta (IL 1-beta) (**e**), the tumour necrosis factor-alfa (**f**), and the nitric oxide synthase 2 (NOS2) (**g**). The analysis revels that NURR1^+/−^ mice show an increase in the gene expression level of HMOX and a reduction of MT2 respect to WT. Mann–Whitney *U* test, *p < 0.05

Microglial markers of cellular damage, apoptosis, cellular response to stress, pro-inflammatory, and neuroprotective effectors were also selected [59]. To evaluate whether apoptotic or cell damage mechanisms are activated in the SN of NURR1^+/−^ mice, as occurs during inflammatory processes, the expression levels of the heme oxygenase 1 (HMOX) and the poly ADP-ribose polymerase family, member 1 (PARP1) gene were measured. NURR1^+/−^ mice show an increased expression level of the HMOX gene (Fig. 4a, * *p* = 0.0485, Mann–Whitney *U* test), but not the PARP1 (Fig. 4b, *p* = 0.0727, Mann–Whitney *U*) compared to WT mice, suggesting that some of such apoptotic or damaging mechanisms in the SN of NURR1^+/−^ mice probably occur.

Among markers of the cellular response to stress specifically activated by an inflammatory stimulus, metallothionein (MT) 1 and 2 were evaluated. NURR1^+/−^ mice showed no differences in the expression of MT1 (Fig. 4c, *p* = 0.9333, Mann–Whitney *U* test), but surprisingly a low level of expression of MT2 (Fig. 4d, * *p* = 0.0485, Mann–Whitney *U* test) compared to WT, indicating a clear difference compared to what happens during an inflammatory process, in which the expression of these genes increases as a stress response.

No significant differences emerge between genotypes in the expression of the pro-inflammatory molecules, such as the interleukin-1-beta (IL 1-beta, Fig. 4e, *p* = 0.5616, Mann–Whitney *U* test), the tumour necrosis factor-alfa (TNF-alfa, Fig. 4f, *p* = 0.1091, Mann–Whitney *U* test), and the nitric oxide synthase 2 (NOS2, Fig. 4g, *p* = 0.1091, Mann–Whitney *U* test), indicating that the SN of NURR1^+/−^ mice is not characterized by a pro-inflammatory milieu.

**Fig. 5:**
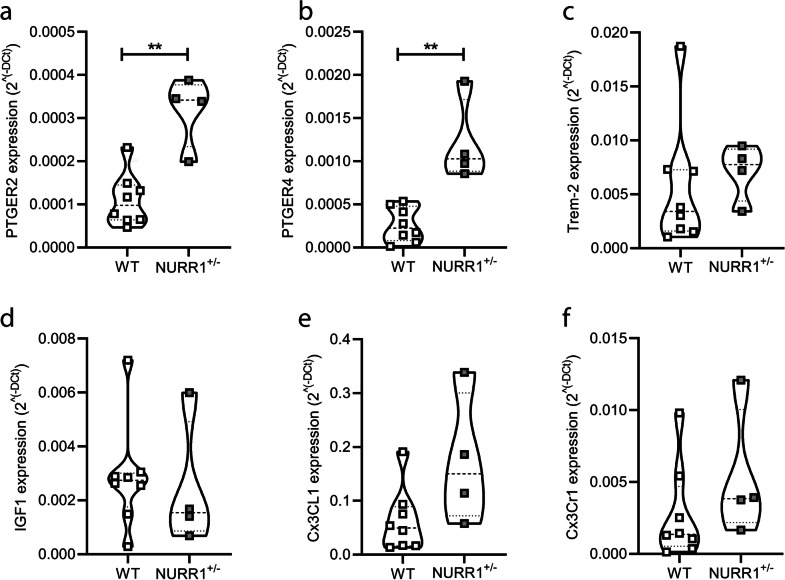
**Expression levels of effector molecules related to the neuroprotective microglial phenotype**. Comparison of gene expression levels in the SN of WT (*n* = 8) and NURR1^+/−^ (*n* = 4) mice of the prostaglandin E receptor 2 (subtype EP2) (PTGER2) (**a**) and receptor 4 (subtype EP4) (PTGER4) (**b**), the triggering receptor expressed on myeloid cells 2 (Trem-2) (**c**), the insulin-like growth factor 1 (IGF1) (**d**), the chemokine (C-X3-C motif) ligand 1 (Cx3CL1) (**e**), and the chemokine (C-X3-C motif) receptor 1 (Cx3Cr1) (**f**). The analysis revels that NURR1^+/−^ mice show an increase in the gene expression level of PTGER2 and PTGER4 respect to WT. Mann–Whitney *U* test, **p < 0.01

Among the markers of neuroprotection associated to the microglial compartment, NURR1^+/−^ mice showed an increased expression level of the prostaglandin E receptor 2 (subtype EP2) (PTGER2, Fig. 5a, ** *p* = 0.0081, Mann–Whitney *U* test) and receptor 4 (subtype EP4) (PTGER4, Fig. 5b, ** *p* = 0.0040, Mann–Whitney *U*) compared to WT littermates. Otherwise, no differences emerged between genotypes in the triggering receptor expressed on myeloid cells 2 (Trem-2, Fig. 5c, *p* = 0.2141, Mann–Whitney *U*), the insulin-like growth factor 1 (IGF1, Fig. 5d, *p* = 0.4606, Mann–Whitney *U*), the chemokine (C-X3-C motif) ligand 1 (Cx3CL1, Fig. 5e, *p* = 0.0727, Mann–Whitney *U*), and the chemokine (C-X3-C motif) receptor 1 (Cx3Cr1, Fig. 5f, *p* = 0.1535, Mann–Whitney *U*).

### *NURR1*^+*/−*^* Mice Show No Alterations in the Redox-Related Parameters Compared to WT Mice*

**Fig. 6:**
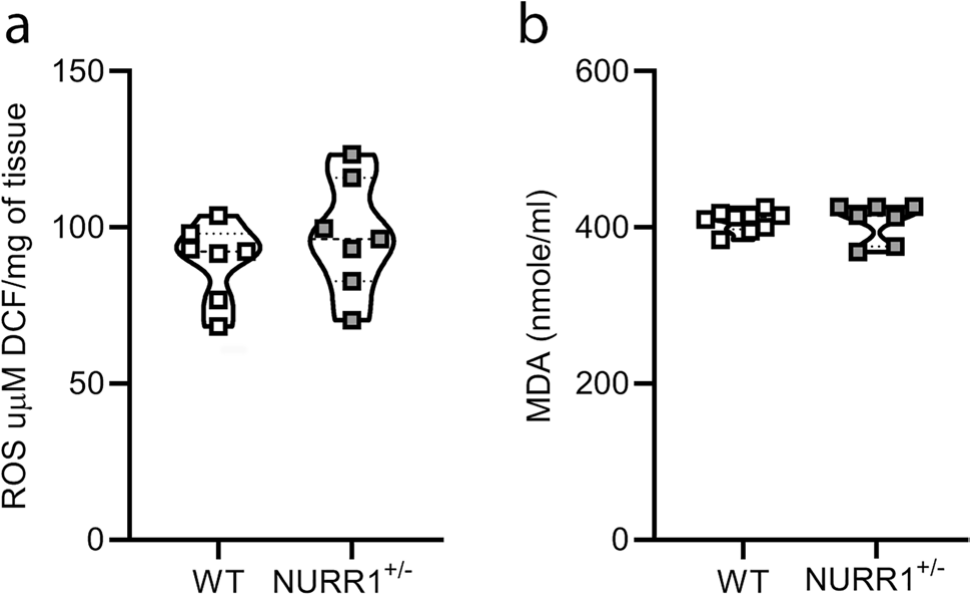
**NURR1**^+/−^** mice show no alterations in the ROS levels and the MDA amount compared to WT mice.** Quantitative analysis of the reactive oxygen species (ROS) levels (**a**), and malondialdehyde (MDA) amount (**b**) in the SN of WT (*n* = 7) and NURR1^+/−^ (*n* = 7) mice revels no differences between genotypes. Mann–Whitney *U* test

In order to investigate possible alterations of redox-related parameters in the SN obtained from NURR1^+/−^ mice, the ROS levels and the MDA amount were investigated. No significant differences emerge between genotypes (Fig. 6a, *p* = 0.3176, Fig. 6b, *p* = 0.5538, Mann–Whitney *U* test).

## Discussion

The present work shows that NURR1 deficiency in male mice determines a reduction in the number of Iba1+ microglial cells specifically in the SN. Such heterozygous NURR1^+/−^ mice are characterized by an increase in locomotion and thus hyperactivity [44–47], even if the number of their mDA neurons and the level of DA are preserved [42, 44, 60]. However, it should be stressed that the activity of mDA neurons in heterozygous NURR1^+/−^ mice has never been investigated, and that homozygous NURR-knockout mice die soon after birth [4], making complete gene deletion studies in mice unachievable.

Former studies reveal that microglia differ in cell number in relation to their regional distribution, and that SN is among the most densely populated areas [61–63]. Previous results propose also, that region-specific differences in the number of microglia induces region-specific susceptibility to damage mediated by microglia [61]. Specifically, when mixed neuron-microglia cultures derived from high-density Iba1+ mesencephalic areas are treated with an inflammatory stimulus, such as lipopolysaccharide (LPS), such cultures become more sensitive with a production of inflammatory factors than hippocampal or cortical cultures [61]. Overall, this suggests that microglia may differently respond to insults depending on their regional distribution, and thus on their basal cell density.

Although microglia-mediated neuroinflammation can induce brain dysfunction in neurological and psychiatric disorders [64, 65], the exact mechanism by which the altered number of the Iba1+ cells may affect the control of psychiatric behaviours is not yet understood. Recent reports demonstrate that even just a time-limited depletion of microglia during embryogenesis has long-term effects on behaviour in adulthood, including the development of hyperactivity [66], as might occur in NURR1^+/−^ mice.

The detailed analysis performed on the microglia cells shows that even if Iba1+ cells of NURR1^+/−^ mice are reduced in number, they are not morphologically characterized by a different state of activation. Overall, these findings might suggest a possible developmental defect or impaired survival of microglia related to NURR1 deficiency, rather than their altered activation state. This concept is supported by the up-regulated expression of HMOX gene in the SN of NURR1^+/−^ mice, which, in microglia is a typical marker of cellular damage and apoptosis [67]. However, further studies will be necessary to evaluate the presence of markers of apoptosis in microglial cells in the SN of NURR1^+/−^ mice. Interestingly, the overexpression of HMOX1 in astrocytes induces a hyperactive behaviour in mice [67], as occurs in NURR1^+/−^ mice.

The SN of NURR1^+/−^ mice is also characterized by a lower level of expression of the MT2, which is a marker of cellular response to stress [68]. Published results also report such decrease in the SN of the pharmacologically induced PD murine model, indicating that the expression and activity of MT are critically involved in the control of neurotoxic mechanisms on mDA neurons [69].

The levels of transcripts of pro-inflammatory molecules, such as IL1-beta, TNF-alpha, and NOS2, are not altered in the SN of NURR1^+/−^ mice. Considering its previously highlighted anti-inflammatory role [24], this might seem quite surprising. However, Saijo and colleagues demonstrated the anti-inflammatory function of NURR1 after administration of a pro-inflammatory stimulus, such as LPS, while the SN of the analysed NURR1^+/−^ mice corresponds to a not-inflamed basal environment. In addition, using heterozygous NURR1^+/−^ mice we are unable to completely delete the gene, and thus probably to fully block its signalling.

Among the evaluated markers of neuroprotection, only PTGER2 and PTGER4 are up-regulated in the SN of NURR1^+/−^ mice. Recently, it has been demonstrated that prostaglandins, which are the common endogenous ligands of such receptors, are also able to directly interact with the LBD of NURR1 to mediate a protective role on mDA neurons in a mouse models of PD [2]. However, the consequences of such increase are unknown and should be further investigated.

Interestingly, no differences in redox-related parameters, such as ROS amount and lipid peroxidation, were detected between WT and NURR1^+/−^ mice. This interesting finding could be interpreted in the light of the significant contribution of NURR1 in the physiological maintenance of the redox state, resulting from the balancing of the expression and functioning of the major oxidative stress sources in the CNS, such as mitochondria [38], with the ones of different ROS-scavengers, including glutathione peroxidase-1 and superoxide dismutase 1 [70, 71]. Moreover, to reduce the inflammatory response, NURR1 has been reported to be negatively associated with NF-κB [72, 73], whose signalling shows a complex interplay with oxidative stress through anti- and pro-oxidant properties depending both from the cell type and the cellular oxidative status [74]. Although in our experimental conditions, the number of Iba1+ microglia cells, playing a key role in the regulation of the redox state in the CNS, was reduced in the SN of NURR1^+/−^mice, we could hypothesize that other glial cells, such as GFAP+ astrocytes, which were, instead, not changed in number, might have contributed to the maintenance of a redox equilibrium. However, at this stage, we cannot totally exclude the existence of a possible link between NURR1 and other major ROS sources in the CNS, such as the nicotinamide adenine dinucleotide phosphate (NADPH) oxidase (NOX) enzymes, particularly expressed in both microglial and astrocytic cells [75]. Thus, further research unravelling this aspect is certainly needed.

In conclusion, this work shows that the changes observed in the number of microglial cells in a region-dependent manner might be involved in the behavioural defects observed in NURR1-deficient mice, such as hyperactivity [44]. However, further studies using NURR1 conditional knockout mice, which allow the specific deletion of NURR1 in microglia, are necessary to demonstrate how NURR1 reduction affects microglial cell density and to determine the exact role of microglia in behavioural impairment.

## Supplementary Information

Below is the link to the electronic supplementary material.

ESM 1 (PNG 569 KB)

Supplementary file1 (TIF 3882 KB)

ESM 2 (PNG 196 KB)

Supplementary file2 (TIF 1490 KB)

ESM 3 (PNG 160 KB)

Supplementary file3 (TIF 1217 KB)

Supplementary file4 (DOCX 13 KB)

## Data Availability

The datasets generated during and/or analyzed during the current study are not publicly available due to privacy/ethical restrictions but are available from the corresponding author upon reasonable request.
