# Supplementary figures and images for: NURR1 Deficiency Is Associated to Altered Microglial Phenotype in Male Mice
Source: Mol Neurobiol. 2025 Mar 8;62(7):8887–99. doi: 10.1007/s12035-025-04787-8 (PMC12208995; doi:10.1007/s12035-025-04787-8)

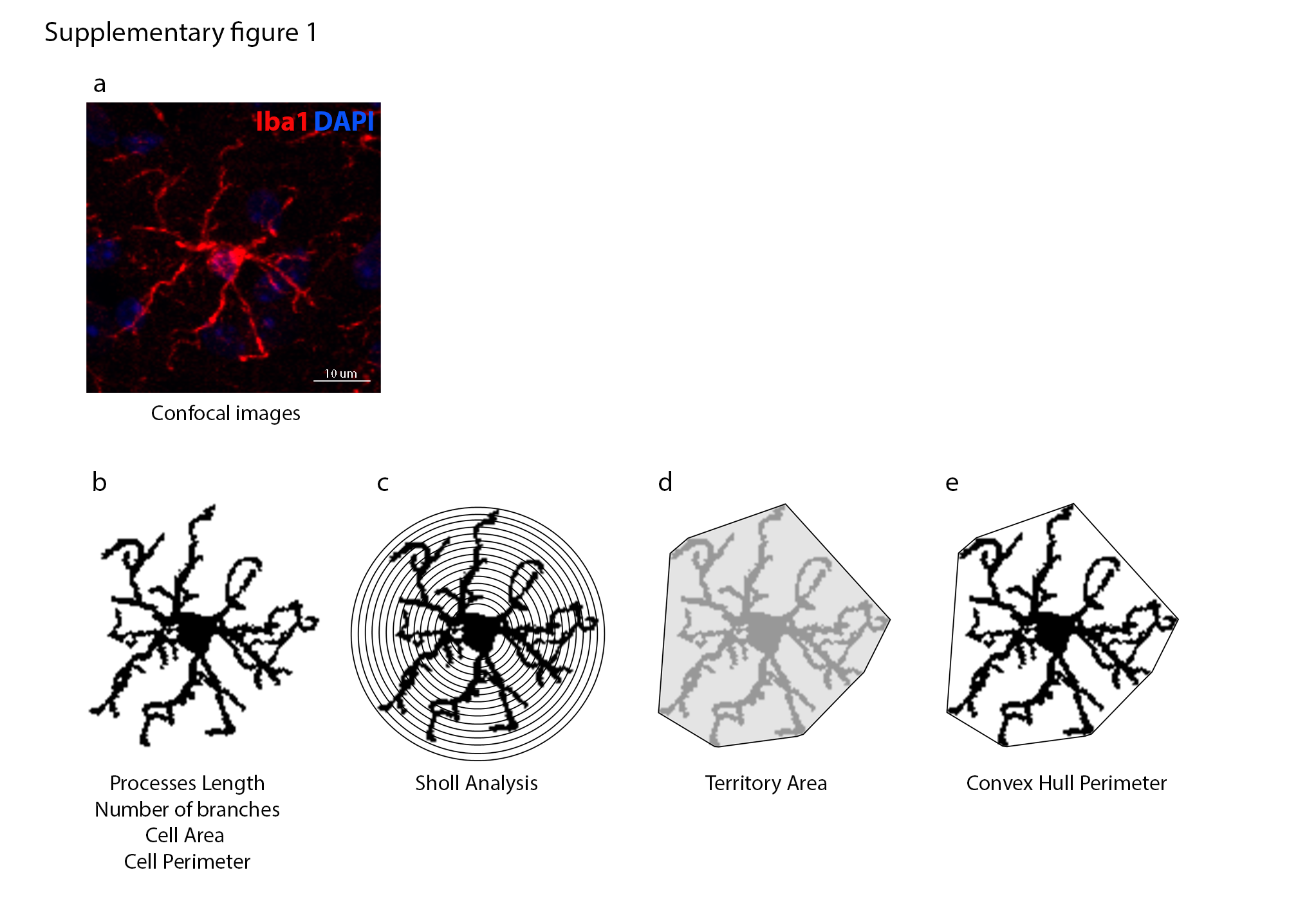

Supplement: Supplementary file 1 — (PNG 569 KB) [file 12035_2025_4787_Fig7_ESM.png]

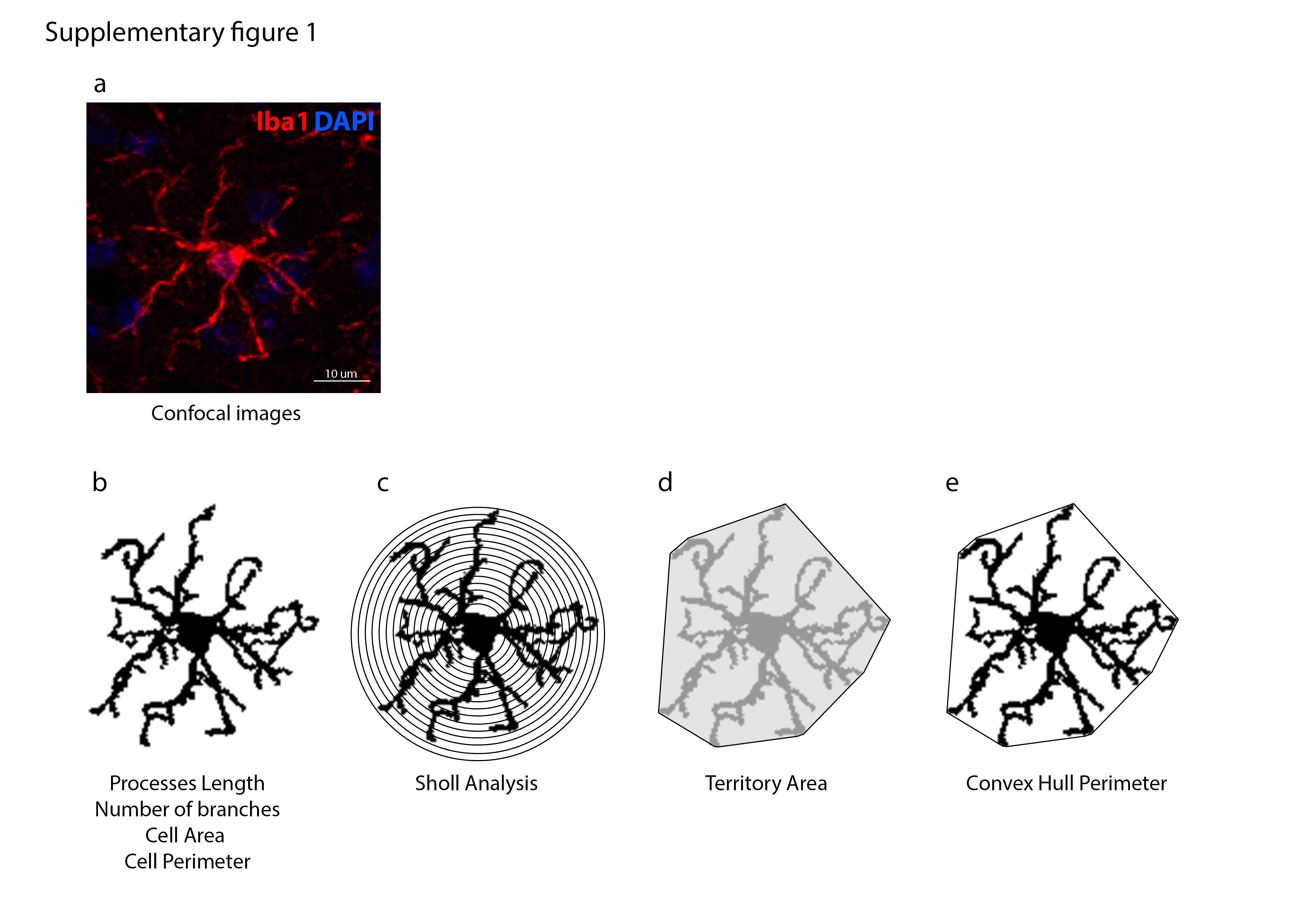

Supplement: Supplementary file 2 — Supplementary file1 (TIF 3882 KB) [file 12035_2025_4787_MOESM1_ESM.tif]

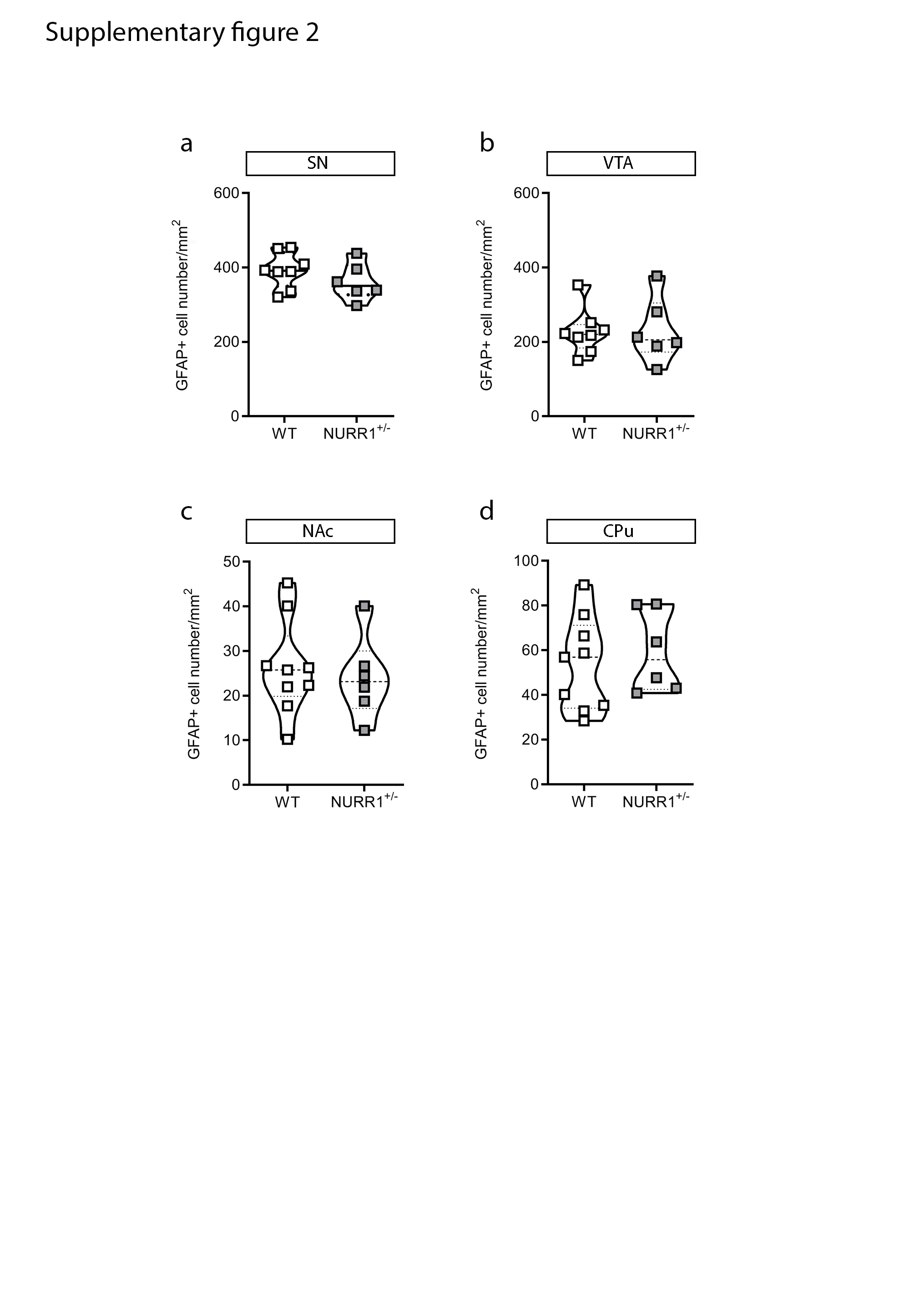

Supplement: Supplementary file 3 — (PNG 196 KB) [file 12035_2025_4787_Fig8_ESM.png]

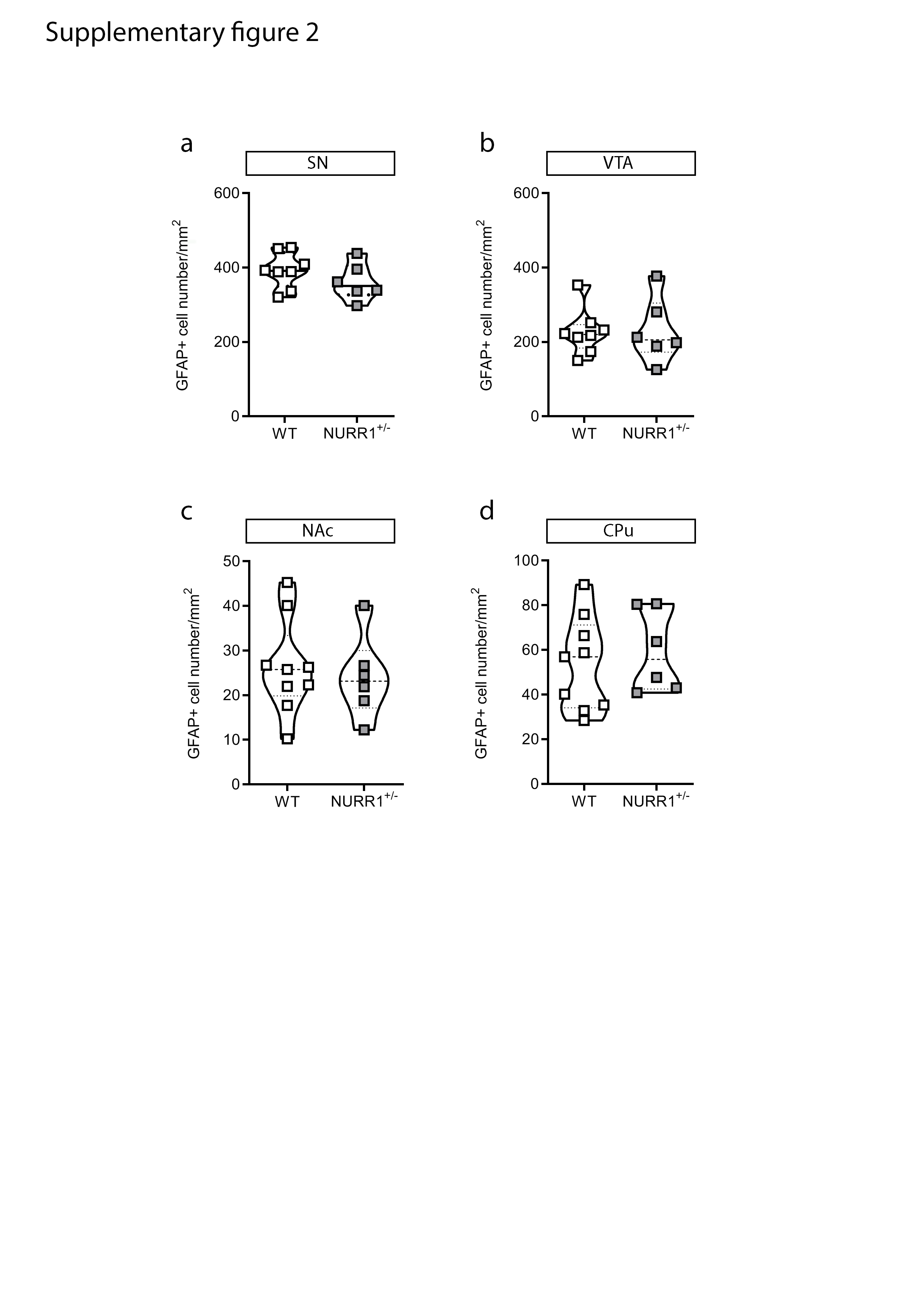

Supplement: Supplementary file 4 — Supplementary file2 (TIF 1490 KB) [file 12035_2025_4787_MOESM2_ESM.tif]

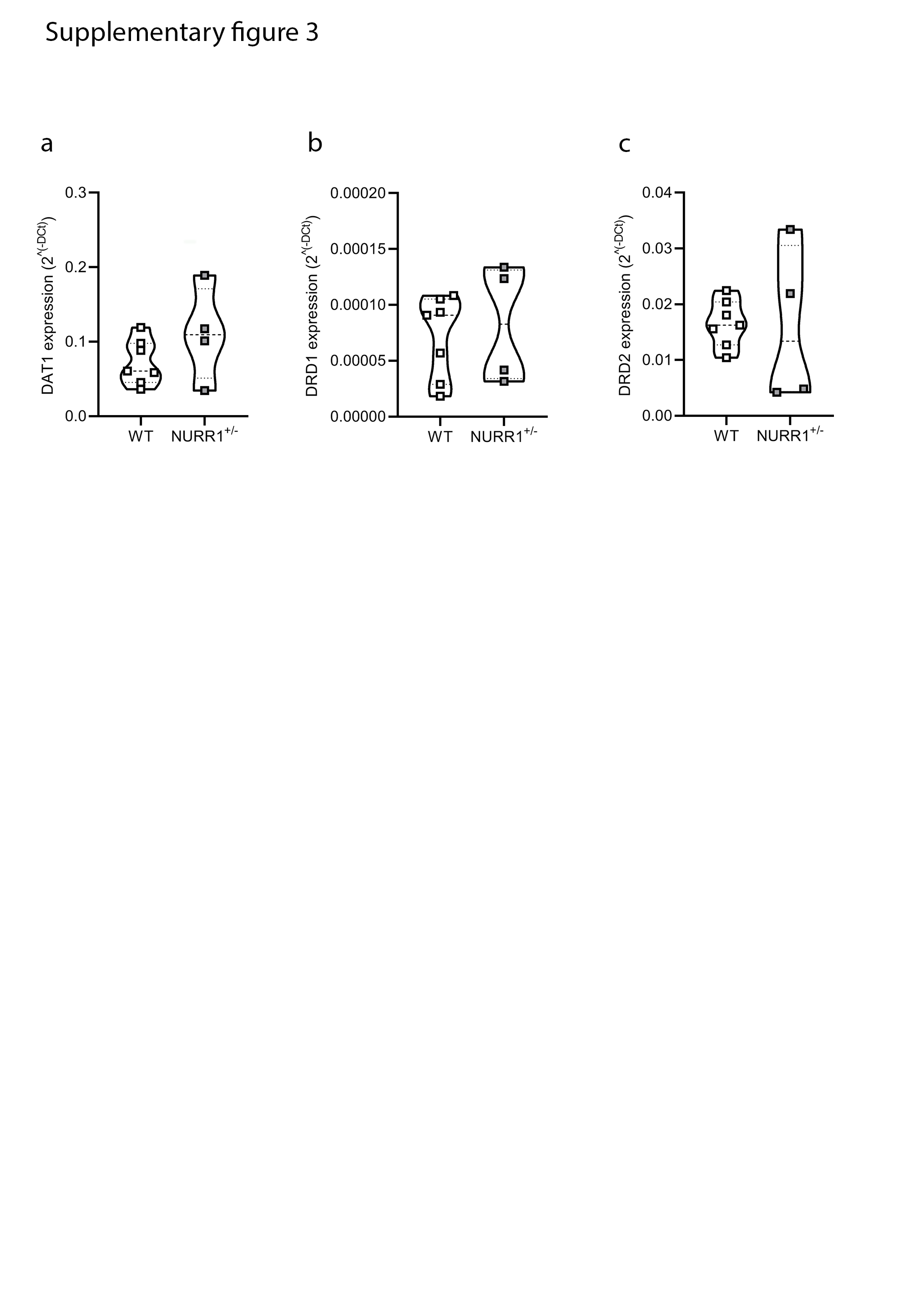

Supplement: Supplementary file 5 — (PNG 160 KB) [file 12035_2025_4787_Fig9_ESM.png]

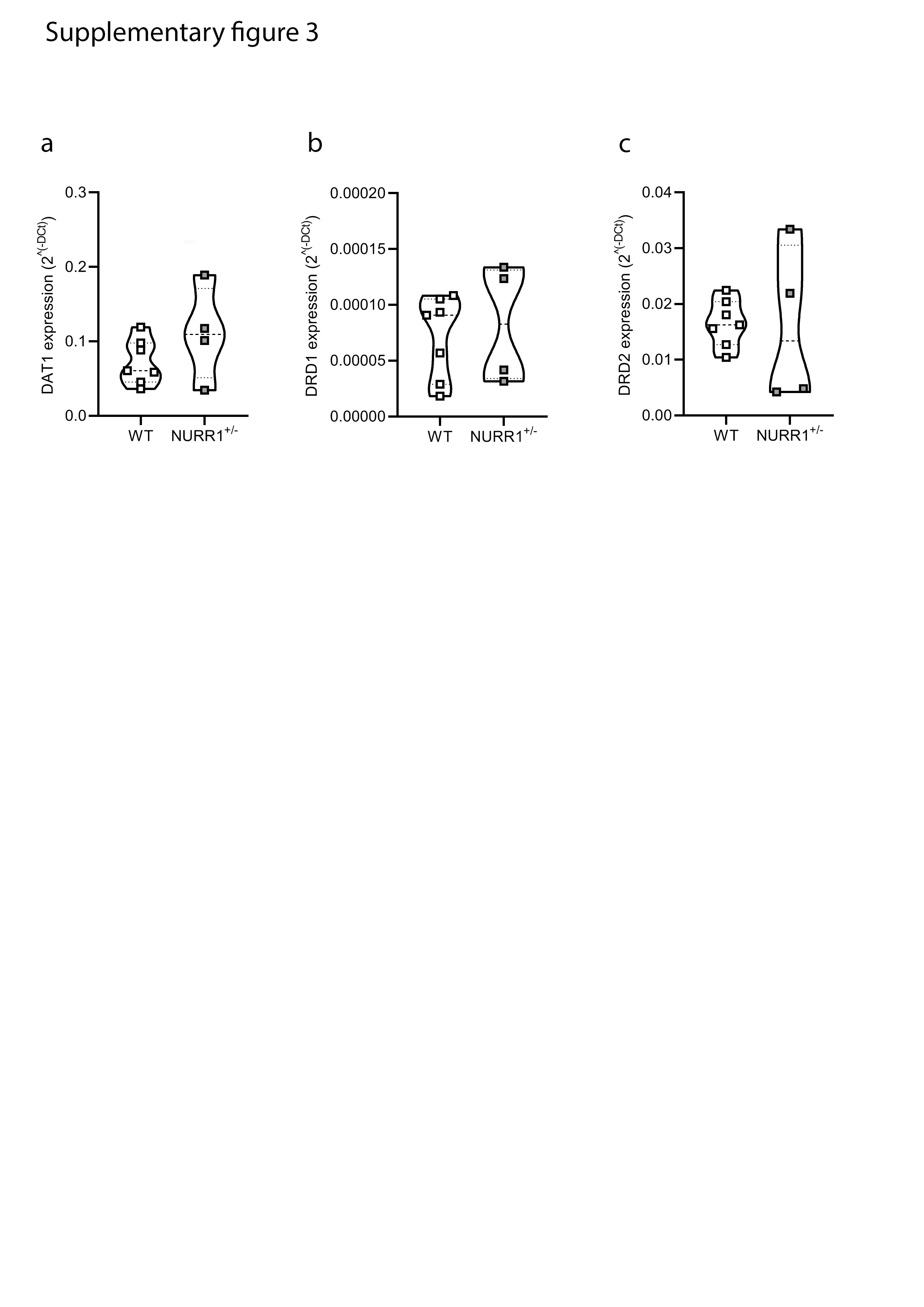

Supplement: Supplementary file 6 — Supplementary file3 (TIF 1217 KB) [file 12035_2025_4787_MOESM3_ESM.tif]
