# Supplementary material for: NURR1 Deficiency Is Associated to Altered Microglial Phenotype in Male Mice
Source: Mol Neurobiol. 2025 Mar 8;62(7):8887–99. doi: 10.1007/s12035-025-04787-8 (PMC12208995; doi:10.1007/s12035-025-04787-8)
Supplement: Supplementary file 7 — Supplementary file4 (DOCX 13 KB) [file 12035_2025_4787_MOESM4_ESM.docx]

**Supplementary materials and methods**

PCR analysis of tail DNA from mice was carried out. Three oligonucleotides (i.e. 5’ GGCACTCCTGTGTCTAGCTGCC, 5’ CTGCCTTGGGAAAAGCGCCTCC and, 3’ CAGCCCTCACAAGTGCGAACAC) designed by Saucedo-Cardenas and colleagues [48] were used in a single PCR. The PCR protocol follows a standard procedure, as indicated below: (I) 4 minutes at 95 °C, (II) 1 minute at 95 °C, (III) 1 minute at 65 °C, (IV) 1 minute at 72 °C (repeat 35 times from (II)), and (V) 5 minutes at 72 °C. The electrophoretic run was performed on Tris-Acetate-EDTA (TAE) buffer 1% agarose gel in ethidium bromide.
